# Adrenocortical carcinoma in a young adult male with chronic urticaria: A case report and literature review

**DOI:** 10.1016/j.ijscr.2019.12.028

**Published:** 2019-12-26

**Authors:** Yasmin Moussa, Mohamad Moussa, Mohamed Abou Chakra

**Affiliations:** a Clinic of Dermatology, Dr. Brinkmann, Schult & Samimi-Fard, Barbarastraße 15, 45964 Gladbeck, Germany; b Head of Urology Department, Zahra Hospital, University Medical Center, Lebanese University, Beirut, Lebanon; c Faculty of Medicine, Department of Urology, Lebanese University, Beirut, Lebanon

**Keywords:** Adrenocortical carcinoma, Mitotane, Urticaria, Case report

## Abstract

**Background:**

Adrenocortical carcinoma (ACC) is a rare endocrine malignancy, often with an unfavorable prognosis. Radical adrenalectomy is the gold standard of treatment of localized disease.

**Case description:**

We report a case of a 23-year-old male patient who presented with persistent left flank pain and urticaria for 3 months. Imaging studies confirmed the presence of a large left adrenal mass with malignant features. The biochemical workup was unremarkable. Open left radical adrenalectomy was performed, the final pathologic examination showed ACC with negative surgical margins. The patient remained disease-free for eighteen months period of follow up after surgery.

**Discussion:**

ACC is a rare neoplasm with poor prognosis and with an incidence of one in one million population. There is a slight female predilection. The ACC may be functional with a clinically pure endocrine syndrome like Cushing syndrome. Most of patients with ACC present with symptoms and signs of hormonal secretion. Adrenal computed tomography (CT) scanning and magnetic resonance imaging (MRI) are the imaging studies of choice in ACC. When feasible, total resection remains the treatment of choice for the definitive treatment of ACC. The benefit of the use of mitotane as an adjuvant treatment has been considered controversial. Adjuvant mitotane significantly decreases the recurrence and mortality rate after resection of ACC in patients without distant metastasis as proved by some studies, but these findings need further validation.

**Conclusion:**

ACC is a rare neoplasm characterized by a high risk of recurrence after surgical resection.

## Highlights

• Adrenocortical carcinoma (ACC) is a rare, aggressive tumor arising from the adrenal cortex.
• Recognition of the typical clinical, biochemical, and imaging findings is crucial for rapid diagnosis.
• Complete surgical resection is currently the curative treatment of localized ACC.
• Adjuvant mitotane may prolong recurrence-free survival in patients with radically resected ACC.

## 1 Introduction

Adrenocortical carcinomas (ACCs) are rare tumors with an estimated annual incidence of 0.7–2 cases per year and a worldwide prevalence of 4–12 cases per million/year. Most ACCs cause hypersecretion of steroids including glucocorticoids and androgens. ACC patients have a very poor prognosis with a 5-year overall survival below 30% in most series [1]. In a study of 105 patients diagnosed with ACC, the average duration of symptoms before the diagnosis was 8.7 months. At the time of diagnosis, 68 percent of the patients had endocrine symptoms, and 30 percent of them had distant metastases. Hormonal studies showed that 79 percent of the tumors were functional [2].

Patients usually present with hormonal hypersecretion signs (e.g. virilization, Cushing’s syndrome) or mass effect. Tumors typically appear inhomogeneous in both computerized tomography and magnetic resonance imaging with necrosis and irregular borders and differ from benign adenomas by their low fat content [3].

In patients with suspected localized ACC, a thorough endocrine and imaging workup is followed by complete resection of the tumor by an expert surgeon. In patients with advanced disease at presentation or recurrence not amenable to complete resection, mitotane alone or in combination with cytotoxic drugs is the treatment of choice [4].

This work has been reported in accordance with the SCARE criteria [5].

## 2 Case report

A 23 years old male patient, Know to have poorly controlled diabetes mellitus type 1(last HBA1C of 7% (normal: 4%–5.6%) presented to the outpatient clinic with 3 months history of left flank pain. His pain was associated with a weight loss of 8 kg. The patient denied any recent sexually transmitted disease or genitourinary trauma. He is a non-smoker and non-alcoholic.

**Fig. 1:**
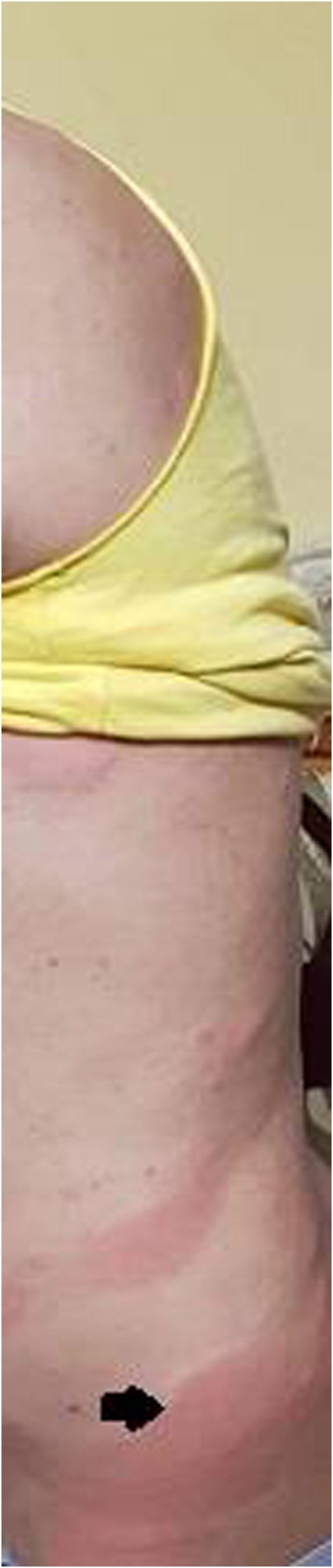
Erythematous, oedematous papules on the back.

On presentation, his temperature was 37 °C and the vital signs were within normal range. Physical examination revealed, severe left Costovertebral angle tenderness. Skin observation revealed blancheable, erythematous, oedematous papules or ‘weals’ identified on the back and buttock (Fig. 1). His urticaria began to appear 3 months ago and it was refractory to anti-histamine and steroids. The patient had no history of allergic symptoms such as angioedema or wheezing. The testicular and digital rectal examinations were normal. No dysuria or frequency or hematuria.

Laboratory examination revealed WBC of 5000/mm3 with normal eosinophils, CRP of 2 mg/L, random blood sugar 200 mg/dl. Urine analysis showed 1–2 WBC per high power field. Serum creatinine was 1.7 mg/dL. Skin patch test was negative.

**Fig. 2:**
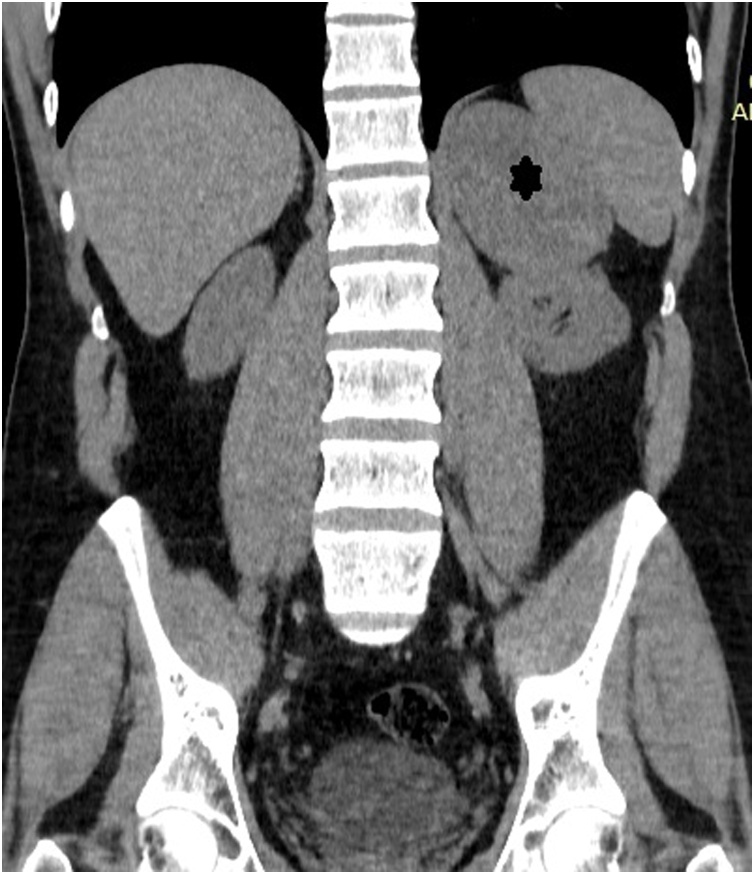
CT abdomen/pelvis revealing a 8 × 5 cm left adrenal mass(Asterick).

**Fig. 3:**
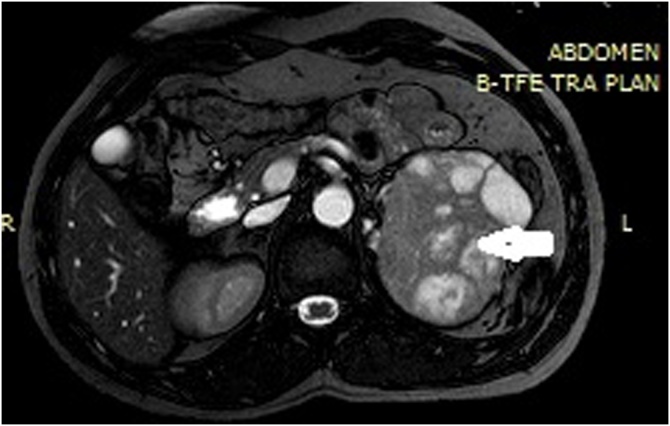
Axial sections of MRI abdomen showed the hetrogenous mass in the left adrenal gland(arrow).

Computed tomography (CT) scan of the abdomen and pelvis without IV contrast showed an 8 × 5 cm mass in the left adrenal gland (Fig. 2). MRI of the abdomen with adrenal protocol was then performed, showed an 8 × 5 cm enhancing left adrenal mass (Fig. 3). It revealed heterogenous predominantly high T2 signal intensity and heterogenous low T1 signal intensity with a differential diagnosis of adrenal carcinoma, and metastatic disease.

Biochemical workup (cortisol, aldosterone, normetanephrines, total metanephrines, and urine metanephrines) was unremarkable aside from plasma dehydroepiandrosterone-sulphate (DHEA-S) level was 120.6 mg/dL (normal 280–640 μg/dL). A metastatic workup including a Computed tomography (CT) of the chest and a bone scan was negative for metastases.

Surgical resection was recommended and the patient underwent open left adrenalectomy. There was no evidence of gross invasion of the surrounding structure intraoperatively.

**Fig. 4:**
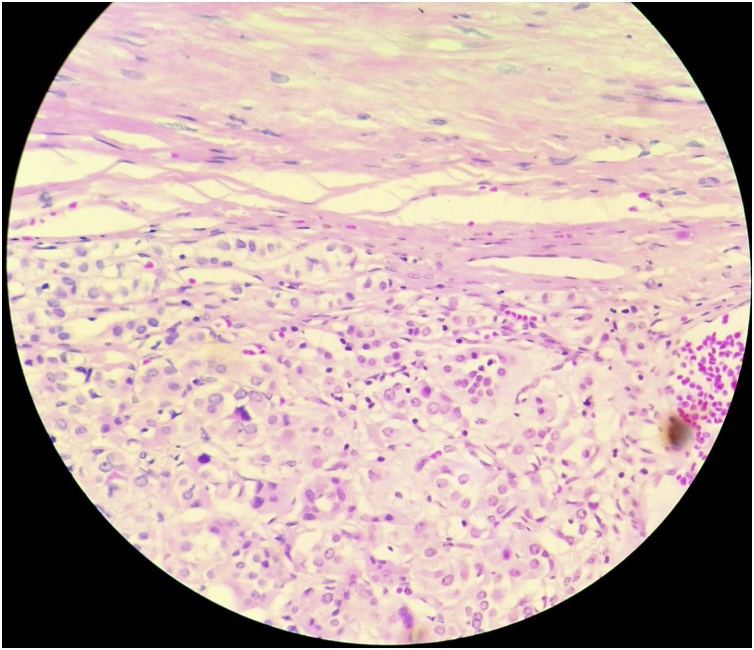
Histological examination, neoplastic cells nuclear pleomorphism.

Surgical pathology confirmed the diagnosis of adrenocortical carcinoma, stage II (Fig. 4). Surgical margins were negative. This case was discussed at a tumor board meeting. Many members of the tumor board mentioned that the potential benefit of postoperative adjuvant therapy with mitotane is still a debatable issue. The discussion focused on the absence of prospective randomized trials comparing treatment with mitotane vs. a watchful waiting strategy in localized ACC after surgery. Also, there was no improvement noted in recurrence-free survival or overall survival in cases receiving mitotane as adjuvant therapy in some trials. At the end, adjuvant treatment was not recommended to this case.

The patient was discharged 3 days post surgery. He was seen in the outpatient clinic 4 weeks after discharge and he had marked improvement with the resolution of his urticaria. The clinical and radiologic follow-up with thoraco-abdominopelvic CT for 6, 12 and 18months showed no signs of local recurrence and distant metastases.

The patient provided a written consent for the publication of this clinical case.

## 3 Discussion

ACC, a rare and aggressive malignancy, accounts for up to 14% of adrenal incidentalomas. It has a bimodal age distribution with a peak in the first decade and a peak in adulthood in the fourth and fifth decades. Women are most frequently affected. The most important predictors of survival in these patients are tumor grade, tumor stage, and surgical resection [6].

Several genes have been implicated as tumor drivers in sporadic ACC, including mutations in insulin-like growth factor 2 (IGF2), β-catenin (CTNNB1 or ZNRF3), and TP53. Other germline variants of some of the same genes identified to be drivers of sporadic ACC are also associated with familial tumor syndromes characterized by ACC, including Beckwith-Wiedemann syndrome (BWS), familial adenomatous polyposis (FAP), and Li-Fraumeni syndrome [7].

Most patients (40–60%) present with steroid hormone excess (glucocorticoids, mineralocorticoids, androgens) or abdominal mass effects (30%), but 15–20% of patients with ACC are initially diagnosed incidentally [8].

Contrast-enhanced CT or MRI is the diagnostic imaging modality of choice for initial imaging and staging as well as for follow-up. The typical appearance of ACC on unenhanced CT is a large, inhomogeneous but well-defined suprarenal mass that displaces adjacent structures as it grows. Regions of low attenuation correspond pathologically to necrosis. ACC is typically heterogeneous in signal intensity on MRI because of the presence of hemorrhage and/or necrosis on T1-weighted imaging, ACC is typically isointense or slightly hypointense to normal liver parenchyma, whereas, on T2-weighted imaging, ACC is usually hyperintense to liver parenchyma [9].

The European Network for the Study of Adrenal Tumors (ENSAT) classification, has been proposed. In this system, stage I and stage II as strictly localized tumors with a size of ≤5 or >5 cm. Stage III ACC is defined by the presence of positive lymph nodes, infiltration of surrounding tissue, or tumor thrombus in the vena cava and/or renal vein; and stage IV ACC is restricted to patients with distant metastasis [10].

Currently, the only curative approach to ACC is complete tumor resection. Adjuvant therapies aim to decrease the chance of recurrence [8]. Laparoscopic resection of adrenal tumors that may be ACC remains controversial. In a study conducted by Gonzalez RJ et al., where among 170 patients with ACC, 153 patients underwent open anterior adrenalectomy, 6 underwent laparoscopic adrenalectomy, 1 was treated via an open flank approach, and 10 had no operation. At a median follow-up of 28 months, all 6 patients who underwent laparoscopic resection of ACC had recurrences, and peritoneal carcinomatosis [11].

ACC is a rare neoplasm characterized by a high risk of recurrence after radical resection. Whether the use of mitotane is beneficial or not as an adjuvant treatment has been controversial. A large retrospective study conducted by Terzolo M et al., suggests a benefit associated with the use of adjuvant mitotane therapy after radical resection of adrenocortical carcinoma [12]. Considering this as a retrospective study, it has multiple limitations. Mitotane therapy in these settings needs further demonstration from prospective controlled trials.

Adrenolytic therapy with mitotane administered alone or in combination with other antineoplastic agents is the primary treatment for patients with advanced ACC [13]. Recently the FIRM-ACT trial included 304 patients with metastatic ACC and compared the association of mitotane with etoposide-cisplatin-doxorubicine (M-EDP) with mitotane-streptozotocin (M-Sz) as a first-line or second-line treatment. It concludes that the Rates of response and progression-free survival were significantly better with EDP plus mitotane than with streptozocin plus mitotane as first-line therapy, with similar rates of toxic events, although there was no significant difference in overall survival [14].

Insulin-like growth factor 1 receptor signaling through upregulation of the stimulatory ligand IGF-II has been implicated in the pathogenesis of ACC. A study investigated figtilimumab, a fully human monoclonal antibody directed toward IGF-1R in patients with refractory adrenocortical carcinoma. Eight of 14 patients had a stable disease as their best response [15]. Although molecular targeted therapy based on basic research findings is ongoing, more trials are needed to show definitive effectiveness.

We present a rare case of ACC in a young patient with nonfunctioning tumor. We also proved that that complete surgical resection (R0 excision) offers the best chance for long-term survival in patients with stage II ACC. In our case, there was no evidence of recurrent or metastatic lesions during the follow-up period of 18 months. We also demonstrated the possible association of the malignant disease with chronic urticaria. Dehydroepiandrosterone and its sulfate derivative (DHEA-S) appear to have regulatory effects on immune homeostasis.

Patient’s urticaria may be related to the decreased serum concentration of DHEA-S level. There are several hypothetical explanations of the phenomenon of lower circulating concentration of DHEA-S in chronic urticaria patients. It has been suggested that during chronic inflammatory response and severe stress conditions, a hormonal shift may occur in the adrenal steroid production into the direction of cortisol relative to DHEA-S, which is probably necessary to achieve adequate cortisol level at the expense of adrenal androgens. However, current knowledge prevents answering whether lower circulating DHEA-S concentration is a primary phenomenon or just an accompanying one which appears as a response of different systems to the course of the illness [16].

## 4 Conclusion

ACC is an uncommon malignancy with high recurrence and mortality rates. ACC is often diagnosed at an advanced stage. A multidisciplinary team approach and discussion can provide the best management and prognosis for ACC diagnosed patients.

## Funding source

No funding

## Ethical approval

Ethical approval is not required by our institution.

## Consent

Written informed consent was obtained from the patient for publication of this case report and accompanying images.

## Author contribution

Yasmin Moussa, Mohamed Abou Chakra, Mohamad Moussa: Case report design.

Yasmin Moussa, Mohamed Abou Chakra, Mohamad Moussa: Manuscript preparation.

Mohamed Abou Chakra, Mohamad Moussa: Followed up the patient and revised the manuscript.

Yasmin Moussa, Mohamed Abou Chakra, Mohamad Moussa: Approved the final manuscript.

## Registration of research studies

Not applicable, case report.

## Guarantor

Mohamed Abou chakra.

## Provenance and peer review

Not commissioned, externally peer-reviewed.

## Declaration of Competing Interest

None.
